# Impact of prolonged digital device use on acquired comitant esotropia: ACE-DD study 2

**DOI:** 10.1007/s10384-025-01171-w

**Published:** 2025-03-17

**Authors:** Hirohito Iimori, Noriko Nishikawa, Sachiko Nishina, Tomoyo Yoshida, Takafumi Mori, Osamu Hieda, Akiko Hikoya, Miwa Komori, Shion Hayashi, Takashi Negishi, Toshiaki Goseki, Yoshiko Sugiyama, Akiko Kimura, Takeshi Morimoto, Yukiko Shimizu, Tamami Shimizu, Yoshimi Yokoyama, Hiroko Suzuki, Sadao Suzuki, Noriyuki Azuma, Miho Sato

**Affiliations:** 1 Department of Ophthalmology, Hamamatsu University School of Medicine, 1-20-1 Handayama, Chuo-ku, Hamamatsu, Shizuka 431-3192 Japan; 2 Department of Ophthalmology, Ehime University, Toon, Japan; 3 Department of Ophthalmology, Asahikawa Medical University, Asahikawa, Japan; 4 Division of Ophthalmology, National Center for Child Health and Development, Setagaya-ku, Tokyo, Japan; 5 Department of Ophthalmology, Fukushima Medical University, Fukushima, Japan; 6 Department of Ophthalmology, Kyoto Prefectural University of Medicine, Kyoto, Japan; 7 Department of Ophthalmology, Faculty of Medicine, Yamagata University, Yamagata, Japan; 8 Department of Ophthalmology, Faculty of Medicine, Juntendo University, Bunkyo-ku, Tokyo, Japan; 9 Department of Ophthalmology, International University of Health and Welfare, Atami Hospital, Atami, Japan; 10 Department of Ophthalmology, Kanagawa Dental University, Yokohama Clinic, Yokohama, Japan; 11 Department of Ophthalmology, Kanazawa University, Kanazawa, Japan; 12 Department of Ophthalmology, Hyogo Medical University, Nishinomiya, Japan; 13 Department of Ophthalmology, Osaka University Graduate School of Medicine, Suita, Japan; 14 Department of Ophthalmology, Saneikai Tsukazaki Hospital, Himeji, Japan; 15 Department of Ophthalmology, Saga University, Saga, Japan; 16 Department of Ophthalmology, Japan Community Healthcare Organization Chukyo Hospital, Nagoya, Japan; 17 Department of Public Health, Nagoya City University Graduate School of Medical Sciences, Nagoya, Japan; 18 Medical Research Institute, Tokyo Medical and Dental University, Bunkyo-ku, Tokyo, Japan

**Keywords:** Smartphone, Acquired esotropia, Child, Young adult, Digital device

## Abstract

**Purpose:**

To investigate changes in strabismus angles in children and young adult patients with recent onset of constant acquired comitant esotropia (ACE) following instructions on hand-held digital devices (DD).

**Study design:**

Prospective multicenter non-randomized interventional study.

**Methods:**

This study included subjects aged 5–35 years who developed ACE within 1 year. Subjects were divided into two groups: (i) subjects who used DD for an average of ≥120 minutes/day for junior high-school students and older and ≥60 minutes/day for primary-school students and younger (DD+); and (ii) subjects who used DD for less than that (DD-) based on the questionnaire at the study’s start. During the initial visit, glasses were prescribed when necessary; verbal instructions on DD use including time reduction and viewing-distance elongation were provided. For each group, strabismus angles at the initial visit and at 3 months were compared. Cure was defined as esophoria within 8 prism diopters without symptoms.

**Results:**

In total, 181 cases were investigated. At baseline, strabismus angles in DD+ and DD- groups were 23±14 PD and 25±15 PD at near and 25±13 PD and 27±14 PD at distance, respectively. At 3 months, they were 22±16 PD and 25±15 PD at near and 23±14 PD and 27±13 PD at distance, respectively. Only in the DD+ group, reduction in strabismus angle was observed, but this was not clinically significant. Ten and 1 subjects in DD+ and DD- groups were cured.

**Conclusion:**

Although changes in strabismus angles were not large enough, DD use instructions were beneficial for some ACE subjects.

**Supplementary Information:**

The online version contains supplementary material available at 10.1007/s10384-025-01171-w.

## Introduction

In recent years, an association has been reported between excessive usage of digital devices such as smartphones and developing acute acquired comitant esotropia (AACE) [1, 2]. The time spent using digital devices has increased due to restrictions on leaving home and remote working due to COVID-19 [3], and this tendency has persisted after the pandemic. On average, the daily use of digital devices among Japanese junior and senior high school students is more than four hours and increasing [4]. Therefore, the increasing number of patients with AACE related to excessive use of digital devices remains a concern [5–7]. Some reports suggest that reducing digital device usage time cures or improves esotropia. Meanwhile, others report cases that were not cured, and the relationship between digital device usage and esotropia remains controversial [1, 2, 8, 9].

To our knowledge, only case reports and retrospective studies conducted at a single institution are reported [10] that excessive use of digital devices may be one cause of AACE. Owing to their retrospective nature, some cases had a subacute or intermittent onset of esotropia rather than an acute onset. We conducted a nationwide prospective multicenter observational study on both acute and subacute onset of acquired comitant esotropia (ACE) and digital device (DD) use in Japan (ACE-DD Study 1 [11]). In the previous study, we analyzed 194 subjects with ACE (mean age, 16.8 years) from 55 institutions, with a focus on handheld DDs such as smartphones, tablets and handheld video games (TV and computers not included). Varying clinical features were also revealed among three age groups. The child group (5-12 years old) spent the least amount of time using DD (159 minutes) compared to adolescents (13-18 years old, 210 minutes) and young adults (19-35 years, 267 min/workday), *p*<0.05. The child group also had the largest strabismus angle (mean strabismus angle: near, 30, 22, 18 PD, *p*<0.01; distance, 28, 26, 21 PD, *p*<0.05). These findings suggest that the influence of DD usage on the onset of ACE may vary with age.

We hypothesized that, if inappropriate use of digital devices causes esotropia, providing and following instructions on appropriate DD usage may improve the strabismus. In this study, we provided instructions on DD usage and compared DD usage time, viewing distance, and strabismus angle before and 3 months after the instructions. To access the effect of the instructions, we divided the subjects into two groups; DD over-use group (DD+) and non-overuse group (DD-).

## Study design

Prospective, multicenter, non-randomized interventional study.

## Subjects and methods

The registry criteria for subjects were similar to the ACE-DD study 1 [11]. Shortly, subjects were defined as with diplopia or esotropia occurring within 1 year of the initial visit without intervention of strabismus surgery and aged 5–35 years at the initial visit. There were no neurological symptoms such as abduction limitation, optic nerve swelling, or headache, and head MRI/CT was performed for confirming the absence of abnormalities whenever possible. Exclusion criteria included a history of head trauma or viral infection that may trigger disease onset, or whenever the investigator considered the subject inappropriate. Subjects with amblyopia, strabismus, history of treatment for other ocular diseases, and visual acuity less than 20/20 were included.

Cycloplegic refraction, strabismus angle using the alternate prism cover test (APCT), and questionnaire on DD use were administered at the study initiation. Upon the initial examination, spectacles were prescribed for hyperopia with a refraction of +3.00 D or higher, low-corrected myopia of 1.00 D or higher, and those not currently wearing any. Prism glasses were prescribed according to the patient’s preference, but no clear criteria were used.

This study included subjects who agreed to be followed up for three months after receiving the DD-related lifestyle guidance described below, without botulinum toxin injections or strabismus surgery for the following three months. Subjects were requested to visit for follow-up at one month and three months later.

At the initial visit, subjects and guardians were instructed verbally to (i) reduce DD use to less than 60 minutes per day for primary school children and younger, and less than 120 minutes per day for junior high school students and older; (ii) maintain a viewing distance of at least 30 cm; (iii) take a 5-minute break every 30 minutes; and (iv) record the daily DD viewing time. These instructions were given to the DD+ group as well as to the DD- group.

### Analyses

Based on the time spent using DD surveyed at the initial visit, subjects were divided into two groups: (i) subjects who used DDs for an average of ≥120 minutes/day for junior high-school students and older and ≥60 minutes/days for primary-school students and younger (DD+); and (ii) subjects who used DDs for less than that (DD-). To date, there is no evidence to define excessive use of digital devices, therefore, in this study we devised our own definitions for the DD+ and DD- groups, drawing on the results of a survey on the internet use among young people conducted by the Cabinet Office [4].

The DD+ and DD- groups were compared for age at the initial visit, equivalent spherical power under cycloplegics, near and distant strabismus angle measurements with APCT (Mann-Whitney U test), and changes in strabismus angle among subjects with an initial prescription or renewal of glasses. In order to study the relationship between the subjects with past history of strabismus/amblyopia and digital device usage, we included the subjects who had been treated for more than 1 year ago. The prevalence of anisometropia, defined as a difference in refraction between the two eyes of 1.50 D or more, was compared between the two groups (Fisher’s exact test).

The strabismus angle and duration of DD use were compared between the DD+ and DD- groups at the initial visit and after three months (Wilcoxon signed-rank test). For the DD+ group, the proportion of subjects who were able to maintain 30 cm from the DD was calculated from the questionnaire, and whether the target viewing distance was maintained at the initial visit and after three months.

Cure was defined as esophoria within eight prism diopters at near and distance without subjective symptoms. The number of subjects who achieved cure was compared across these two groups.

All statistical analyses were performed using EZR [12] (Saitama Medical Center, Jichi Medical University), which is a graphical user interface for R (The R Foundation for Statistical Computing). EZR is an enhanced version of R Commander that is capable of adding statistical functions commonly used in biostatistics. Statistical significance was set at a *p*-value <0.05.

## Ethical approval

This study was approved by the Ethics Committee of Hamamatsu University Medical School (19-091), the Central Ethics Review Board of the National Center for Child Health and Development (2019-016) from the two central research institutes, and the ethical review boards of the individual participating sites. Written informed consent was obtained from each subject, or from their guardian if under 20 years of age. The nature and possible consequences of the study were also explained. This study adhered to the tenets of the Declaration of Helsinki. It began at Hamamatsu University in September 2019, with each site participating after IRB approval was obtained and the last site participating in January 2021. Participation in the registry concluded on 28 December 2021.

## Results

In total, 221 subjects were registered, and cases with missing critical data or missing follow-up (n=40) were excluded. The names of the institutions with patient enrollment and the number of reported cases before the exclusion of subjects for each institution are listed in the Online Resource. Afterward, 181 subjects were analyzed who presented within one year of onset of symptoms and underwent an outcome visit three months after enrollment (Table 1). There were 156 subjects (88 males and 68 females) in the DD+, and 25 subjects (15 males and 10 females) in the DD- groups, with a mean age of 16.4 and 18.4 years, respectively. The age of onset was within a maximum of 1 year from the date of the initial visit.

**Table 1:** Patients’ profile at the initial examination

	DD+ group (n=156)	DD- group (n=25)	*p*-value
	Mean (SD), Median (Range)	Mean (SD), Median (Range)	
Age (years)	16.4 (7.4), 15 (5-35)	18.4 (10.0), 17 (5-35)	0.504
Cycloplegic refraction (D) (SE)			
Right eye	-2.83 (3.51), -2.94 (-16.50 - +4.75)	-2.49 (4.12), -1.88 (-9.75 - +4.75)	0.846
Left eye	-2.70 (3.43), -2.85 (-15.88 - +5.75)	-2.42 (4.11), -2.63 (-9.88 - +5.13)	0.722
Strabismus angle (PD)			
At near	23 (15), 20 (0-70)	25 (15), 30 (1-55)	0.47
At distance	25 (13), 25 (0-70)	27 (14), 30 (6-55)	0.407

	n (%)	n (%)	
History of strabismus/amblyopia	15 (9.6)	3 (12.0)	0.719
Anisometropia	8 (5.1)	5 (20.0)	0.020
Prescription of glasses	42 (26.9)	7 (28.0)	1
Using prism glasses (including 1-month medical examination)	32 (20.5)	6 (24.0)	0.791

DD+ group: patients who used DD for an average of ≥120 minutes/day for junior high-school students and older and ≥60 minutes/day for primary-school students and younger. DD- group: patients who used DD for less than that in the DD+ group

Anisometropia was defined as a difference in refraction between the two eyes of ≥1.50 D

DD digital devices, D diopter, SE spherical equivalent, PD prism diopter, SD standard deviation, n number

Cycloplegic refraction of both eyes, APCT at near and distance, and the percentages of subjects with a history of strabismus and amblyopia were not significantly different between the two groups. The prevalence of anisometropia was significantly higher in the DD- group (*p*=0.02).

In the DD+ group, 10 subjects were prescribed prism glasses before or at the initial visit, and 22 subjects were newly prescribed prism glasses at the 1-month follow-up visit, wherein one patient had increased prism power. In the DD- group, no subjects were prescribed prism glasses before the initial visit; however, six subjects were prescribed prism glasses at the 1-month follow-up visit.

**Fig. 1:**
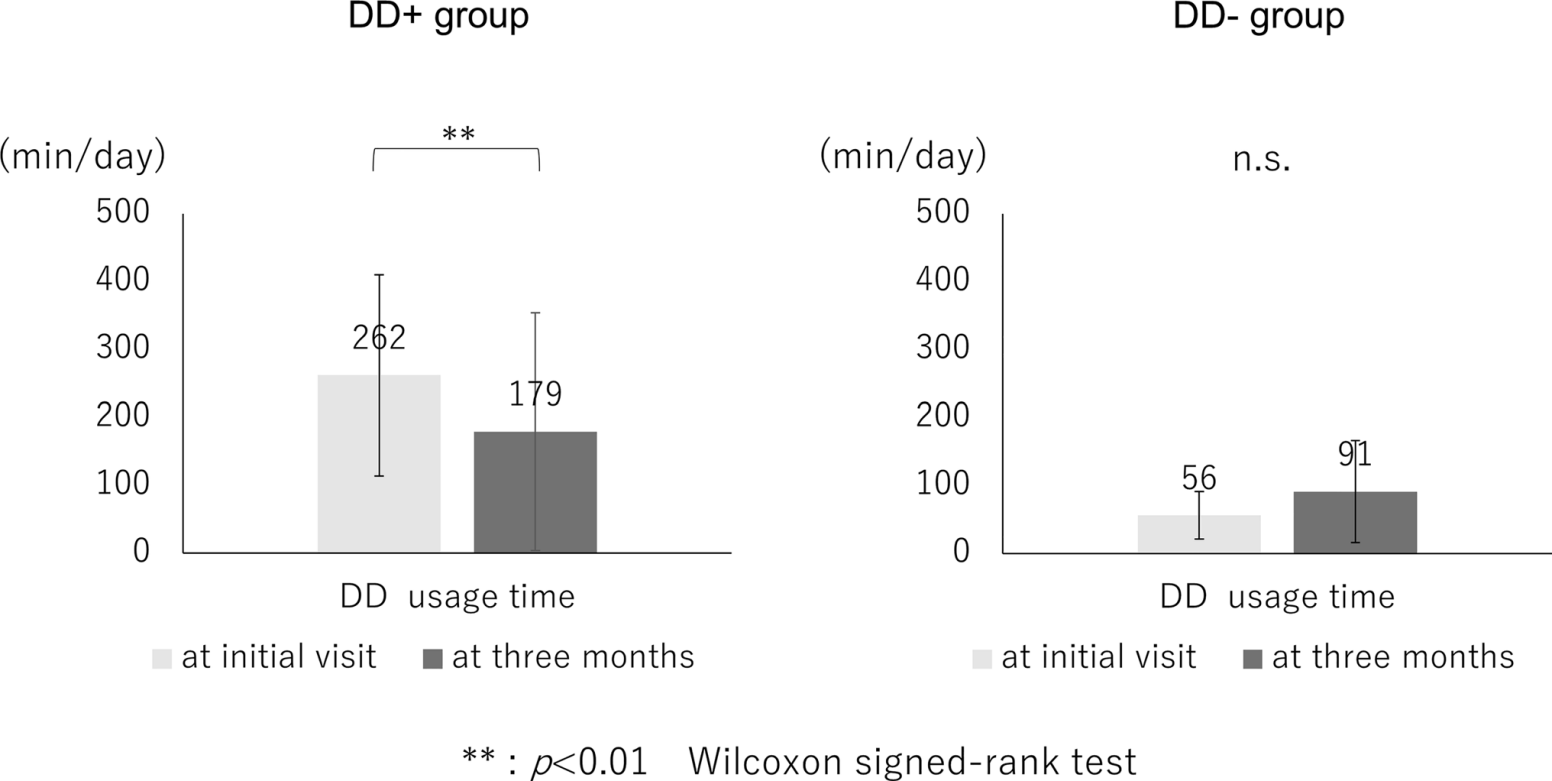
Mean duration of DD use per day in the DD+ and DD- groups at the initial and 3-month visits. The mean durations of DD usage per day at the initial and 3-month visits in the DD+ and DD- groups are shown. In the DD+ group, the time spent using DDs at the 3-month visit was significantly reduced compared to the initial visit. In the DD- group, no significant difference was found between the duration of DD use at the initial and 3-month visits. DD, digital device; n.s., not significant

The mean durations of DD use per day were 262 in the DD+ group at the initial visit and 179 minutes at three months. The durations decreased significantly at three months compared to the initial visit (*p*<0.01). In the DD- group, it was 56 and 91 minutes, respectively, with no significant difference (Fig. 1). The duration of the DD usage time in the DD- group was increased but remained within the instructed time and was less than that of the DD+ group at the 3-month examination (*p*=0.09). In the DD+ group, 16.1% of subjects were able to maintain a viewing distance of more than 30 cm at baseline; however, it increased to 77.0% after instruction.

**Fig. 2:**
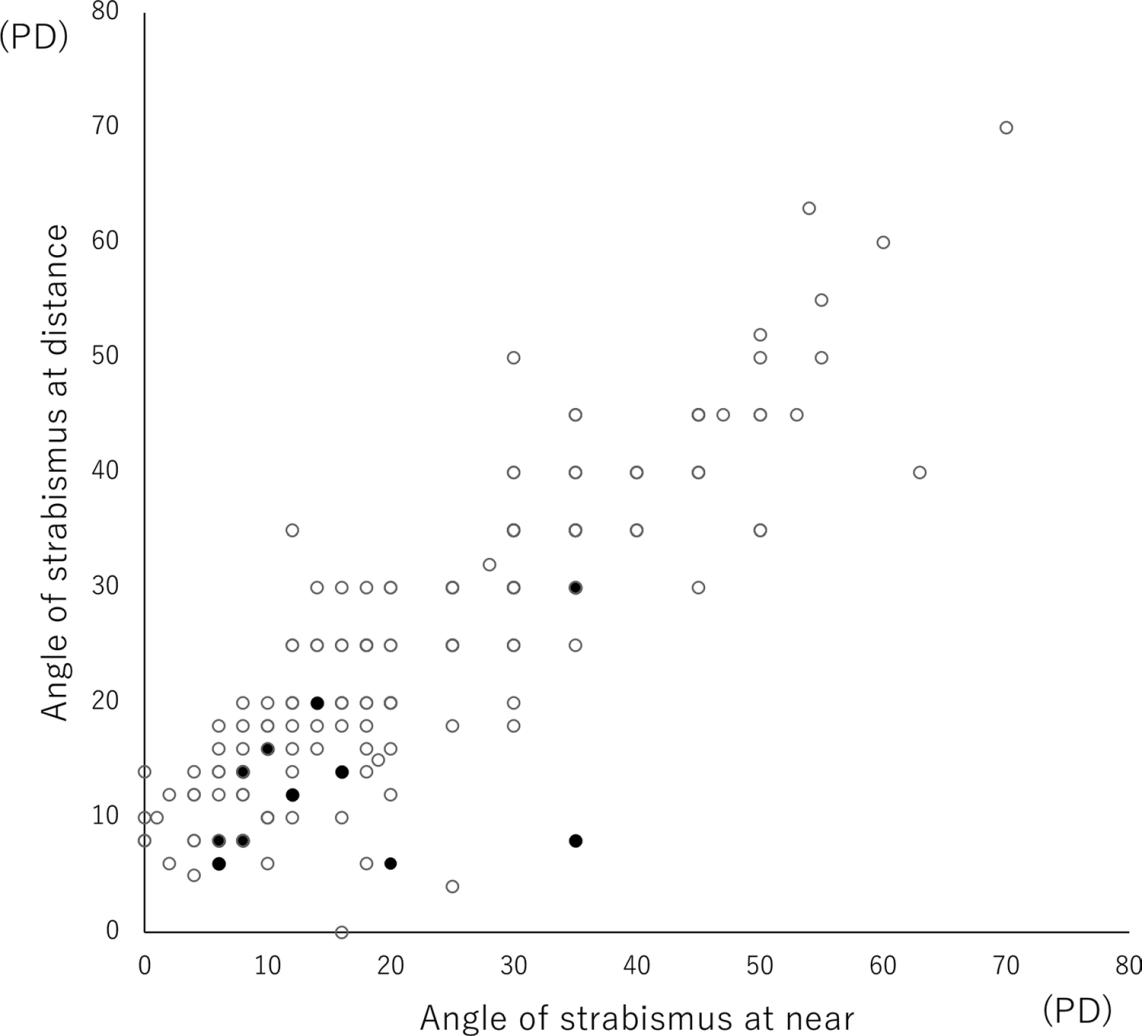
Distribution of strabismus angles at the initial visit in the DD+ group. The distribution of strabismus angles in the DD+ group at the initial visit is shown. Black dots indicate cases that were cured, and white dots indicate cases that were not cured. The mean strabismus angle at the initial visit in cured cases (n=10) was 16±11 PD at near and 13±8 PD at distance. On the other hand, in cases that were not cured (n=143), the mean strabismus angle was 23±15 PD at near and 25±13 PD at distance, and there was a statistically significant difference in the strabismus angle at distance compared with the cured group. (p=0.106 for near, p=0.001 for distance). DD, digital device; PD, prism diopter

The mean strabismus angles in the DD+ group were 23±15 PD and 25±13 PD at the initial visit and 22±16 PD and 23±14 PD at the 3-month visit at near and distance, respectively. The mean strabismus angles in the DD- group at the initial visit were 25±15 PD and 27±14 PD and those at the 3-month visit were 25±15 PD and 27±14 PD at near and distance, respectively. Although there was a statistically significant difference between the angle of strabismus at the first visit and at the 3-month visit in the DD+ group (*p*=0.043 for near,* p*<0.01 for distance), there was no clinically significant difference. Similarly, in the DD- group, no significant changes in APCT for both at near and distance were noted during the study period (*p*>0.05). At the 3-month follow-up, 10 subjects (6.4%) in the DD+ group were cured, and one subject (4.0%) in the DD- group was cured (*p*=1.00). In the DD+ group, the mean strabismus angle at the initial visit in cured cases (n=10) was 16±11 PD at near and 13±8 PD at distance. On the other hand, in cases that were not cured (n=143), the mean strabismus angle was 23±15 PD at near and 25±13 PD at distance, and there was a statistically significant difference in the strabismus angle at distance compared with the cured group. (*p*=0.106 for near, *p*=0.001 for distance) (Figure 2)

Optical corrections for refractive errors were prescribed at baseline in 42 patients (27%) in the DD+ group (mean OD: -2.78±3.92 D) and 7 patients (28%) in the DD- group (mean OD: -3.57±4.82 D) (Table 1). No statistical difference was identified in the spherical power of newly prescribed spectacles between the two groups (*P*=0.549). In the DD+ group, 11 patients and 31 patients received hyperopic and myopic corrections, respectively. No significant reduction in the strabismus angle at the 3-month examination was found. In the DD- group, 2 patients and 5 patients received hyperopic and myopic corrections, respectively. Strabismus angle was not reduced in both cases (Table 2).

**Table 2:** Examination data for patients with prescribed glasses

	DD+ group		DD- group	
	Hyperopia (n=11)	Myopia (n=31)	Hyperopia (n=2)	Myopia (n=5)
	Mean (SD), Median (Range)			
Refraction (D) (SE)	+1.73 (1.39), +1.38 (+0.34 – +4.75)	-4.43 (3.15), -4.06 (-14.8 - -0.13)	+2.44 (1.86), +2.44 (+1.13 - +3.75)	-5.98 (2.96), -6.00 (-9.75 - -1.75)
Angle of strabismus at near (PD)				
At initial visit	27 (15), 25 (10 - 60)	23 (17), 18 (0 - 70)	35.0 (0), 35 (35-35)	26 (11), 30 (12 - 35)
At 3 months	28 (15.2), 30 (4 -50)	22 (18), 18 (0 - 66)	22 (5), 22 (18-25)	27 (16), 28 (6 - 45)
Angle of strabismus at distance (PD)				
At initial visit	27 (15), 25 (10 - 60)	25 (14.3), 20 (8 - 70)	35 (0), 35 (35 - 35)	26 (13), 25 (12 - 45)
At 3 months	25 (13), 25 (0 – 40)	23 (15), 19 (2 – 60)	22 (5), 22 (18 - 25)	28 (13), 30 (8 - 45)

DD+ group: patients who used DD for an average of ≥120 minutes/day for junior high-school students and older and ≥60 minutes/day for primary-school students and younger. DD- group: patients who used DD for less than that in the DD+ group

*DD* digital devices*, D* diopter, *SE* spherical equivalent, *PD* prism diopter, *SD* standard deviation, *n* number, *n.s.* not significant

## Discussion

Upon dividing patients into two groups according to the duration of DD use, there were no differences in age, refraction, or strabismus angle at baseline. In the DD+ group, the duration of DD use was significantly lower after 3 months of instruction, but no clinically significant changes were observed in the strabismus angle. In the DD- group, no significant difference was observed in the strabismus angle between the initial visit and the 3-month visit. However, when looking at individual cases, some cases showed symptoms’ improvement on acquiring the habit of appropriate DD use; thus, it is predicted that there are cases wherein inappropriate use of DD could have a significant impact on the condition. At the same time, there may have been cases in which appropriated refractive correction for either myopia or hyperopia affected eye position. There was a significant difference in the strabismus angle at distance at the initial visit between the cured and non-cured groups. The reason for this is unclear, but it is hypothesized that the remaining fusion ability and the period from onset to treatment are related to the reversibility of the condition; further investigation is needed. Subjects with a smaller strabismus angle had better chances for improvement following DD use instructions.

Various causes of AACE have been suggested [13–15], including mild hyperopia, microtropia, psychological stress, and uncorrected myopia. In this study, some cases of acquired esotropia were not associated with excessive use of DDs (DD- group), and 25% of these cases had anisometropia of 1.50 D or more, suggesting that anisometropia may lead to acquired esotropia independent of excessive use of DDs.

This study had some limitations. First, there was no control group, i.e., a group that did not receive instructions on the use of DD. Second, subjects or their guardians reported the duration and DD use. Hence, the reported time for DD usage might not have been accurate. Third, some patients in the DD+ group might not have followed the instructions, and the changes in strabismus angle owing to DD instructions may not be accurately shown. And lastly, there is some unknown factor as to why some subjects spent much more time watching digital devices.

This prospective non-randomized multicenter study shows that providing instructions for proper DD use was effective in reducing the time spent using DDs and increasing viewing distance. No clinically significant changes were observed in the strabismus angle between the initial and 3-month visits, but in some cases, esotropia was cured. Excessive use of DD may be related to symptoms in some patients; thus, when treating patients with acquired comitant esotropia, it is necessary to first review their DD usage habits and consider providing instructions on appropriate usage. Especially if the strabismus angle is small, it may be worthwhile providing instructions before the angle becomes larger. However, once acquired comitant esotropia develops, there are few cases wherein it can be cured with conservative treatment alone; thus, it is important to identify measures to prevent onset.

## Supplementary Information

Below is the link to the electronic supplementary material.

Supplementary file1 (DOCX 19 KB)
