# Supplementary material for: Impact of prolonged digital device use on acquired comitant esotropia: ACE-DD study 2
Source: Jpn J Ophthalmol. 2025 Mar 17;69(2):166–73. doi: 10.1007/s10384-025-01171-w (PMC12003580; doi:10.1007/s10384-025-01171-w)
Supplement: Supplementary file 1 — Supplementary file1 (DOCX 19 KB) [file 10384_2025_1171_MOESM1_ESM.docx]

**List of participating institutions of 221 cases (number of patients)**

Hamamatsu University School of Medicine (19), Kanagawa Dental University Yokohama Clinic (18), Kyoto Prefectural University of Medicine (15), Kagoshima Miyata Eye Clinic (9), National Center for Child Health and Development (9), Kozaki Eye Clinic (8), Kyorin University School of Medicine (8), Asahikawa Medical University (7), Hyogo medical university (7), Japan Community Healthcare Organization Chukyo Hospital (7), Nagoya University Graduate School of Medicine (7), Juntendo University Nerima Hospital (6), Kagawa Saiseikai Hospital (6), Tokai University School of Medicine (6), Fukushima Medical University (5), Okada Eye Clinic (5), University of Toyama (5), Hidaka Hospital and Heisei Hidaka Clinic (4), Kawasaki Medical School (4), CS eye clinic (3), International University of Health and Welfare Hospital (3), Machida Hospital (3), Nagata Eye Clinic (3), Ochanomizu-Inoue Eye Clinic and Nishikasai-Inouye Eye Hospital (3), Saneikai Tsukazaki Hospital (3), Sugita Eye Hospital (3), Saitama Prefectural Children’s Medical Center (3), Tohoku University Hospital (3), Tottori University (3), Ueoka Eye Clinic (3), University of Tsukuba (3), Ehime University Hospital (2), Fukuoka Children's Hospital (2), Hinokuma Eye Clinic (2), Kobe University Graduate School of Medicine (2), Osaka Women's and Children's Hospital (2), Tokushima University Hospital (2), Chiba Children’s Hospital (1), Heiwa Eye Clinic (1), Ibara City Hospital (1), Iwate medical university (1), Kagoshima University Graduate School of Medical and Dental Sciences (1), Kaiya Eye Clinic (1), Kanazawa University Hospital (1), Kitasato University Hospital (1), Kono Eye Clinic (1), Miki Eye Clinic (1), Nara Medical University (1), Suzuki Eye Clinic Kichikoji (1), Teikyo University School of Medicine (1), Tokyo Metropolitan Hospital Organization Tokyo Metropolitan Tama Medical Center (1), Tokyo Metropolitan Ohtsuka Hospital (1), Totsuka eye clinic (1),

Ueno eye clinic (1), Yamagata University Faculty of Medicine (1)
